# Fabrication of Micro-Scale Gratings by Nanosecond Laser and Its Applications for Deformation Measurements

**DOI:** 10.3390/mi8050136

**Published:** 2017-04-25

**Authors:** Guanbao Yang, Wei He, Jianguo Zhu, Lei Chen

**Affiliations:** 1 Faculty of Civil Engineering and Mechanics, Jiangsu University, Zhenjiang 212013, Jiangsu, China; 18852869269@163.com (G.Y.); chen1573@ujs.edu.cn (L.C.); 2 Department of Mechanics, Tianjin University, Tianjin 300350, China; hewei2008@tju.edu.cn

**Keywords:** grating fabrication, nanosecond laser, geometric phase analysis, deformation measurement

## Abstract

This paper experimentally investigated the fabrication and optimization of micro-scale gratings formed by nanosecond laser etching. The mechanism of nanosecond laser processing and the geometric phase analysis (GPA) are discussed, and the factors influencing the fabrication process including laser energy, laser fluence, and ablation threshold of material, are experimentally studied. In order to eliminate the dependence of the processing parameters on the samples, depositing Al film on a sample before laser processing is proposed for the fabrication of high-quality gratings. The energy of the laser pulse is optimized for clear line etching on Al film considering the distance between adjacent lines of parallel gratings. The optimal energy of the laser pulse is 9.8 μJ, and the optimum fluence is 9.5 J/mm^2^ with the waist radius of the laser beam 25.7 μm. With the optimal parameters, experimental results indicate that the highest frequency of parallel gratings is about 30 lines/mm, with a line width of 29 μm, and the distance between two adjacent laser pulses being of 10 μm. By performing tensile tests, micro-scale gratings fabricated on specimens are experimentally verified. The verification tests prove that the proposed fabrication method for the micro-scale gratings in GPA measurements is reliable and applicable, and the micro-scale gratings can be fabricated in many areas of interest, such as the crack tip, for deformation measurements. Furthermore, the adhesion between the Al film and the tested sample is strong enough so that the pattern sticks well to the sample.

## 1. Introduction

Deformation measurement and analysis are essential for the study of the mechanical properties of materials and structures. With the development of science and technology, measurement methods for multi-scale deformations from macro-scale to micro/nano-scale have drawn great attention [1,2,3]. As a basic optical component, gratings can be used in various optical technologies, such as Moiré interferometry [4], geometric phase analysis (GPA) [5], and many microscopic Moiré methods [6] for measuring surface deformation. Proposed by Hytch et al. in 1998 [7], their work was initially used for measuring and mapping displacement and strain fields with high-resolution electron microscope images. GPA was usually used for local deformation measurements in nanoscale, and the natural atomic lattice was used as a grating [8,9]. Since then, it has been improved by introducing new methods and techniques. Fabricated gratings with frequency from a few tens of lines/mm to thousands of lines/mm were used for macro-scale [10] and micro/nano-scale deformation measurements [11,12]. In addition, various methods were developed to enhance sensitivity and resolution. For example, windowed and discrete Fourier transforms were successfully used to acquire more accurate local phase information [13,14]. Compared to the geometric Moiré method and Moiré interferometry, the GPA method possesses four major advantages: (1) the method is widely applicable to specimen gratings with pitch of atomic lattice up to a few millimeters and the specimen sizes of several nanometers to several millimeters, (2) the field of view can be changed conveniently by adjusting the microscopy magnification, (3) it is a simple test procedure mainly involving the capture of grating images under load, and (4) there are low requirements of test conditions in which vibration isolation is unnecessary. Therefore, it is of fundamental importance to efficiently and reliably fabricate multi-scale gratings. 

As a conventional grating fabrication method developed in the early 1960s [15], the holography lithography technique (HLT) is based on the interference of two coherent beams of light and the exposure of photoresist. The process of HLT is relatively complex, including coating photoresist, exposure, developing, and metal film deposition. The advantage of this technique that it produces gratings very quickly. Usually, the frequency is 1200 lines/mm and the size is a few tens of millimeters. However, the high precision does rely heavily on the optical setups, which cannot be mastered in a short time by learners, which accordingly restrained its wide applications [16,17].

In recent years, researchers have investigated a number of micro-scale fabrication technologies to fabricate gratings, such as the X-ray lithography [18], electron beam lithography (EBL) [19], and focus ion beam milling [20]. For example, the process of EBL uses an exposure source of electron beam, and the grating pattern is formed by a pattern generator. Although high-resolution alignment can be obtained, it is time consuming and has low throughput due to the serial nature of the process. The EBL method is also limited by the size of the processed sample considering the vacuum chamber size. Therefore, the grating made by EBL is more suitable to measure microscopic deformations.

Since the middle 1990s, nanoimprint lithography (NIL) was initially proposed and developed by Chou group [21,22]. The micro- and nano-scale patterns can be manufactured with sub-micron alignment over a large area. A major benefit of NIL is its simple manipulation without complex optics or high-energy radiation sources using a nanoimprint tool. However, the key concerns of NIL are defects, template patterning, and template wearing [23,24].

The laser has been widely used for micro-scale processing due to a very high instantaneous power and small heat-affected zone. The energy of pulse nanosecond laser is very high (the maximum value is up to 1 J), and recently it has been used to fabricate microstructures directly on the surface of materials such as metals, semiconductors, and ceramics [25]. Moreover, the nanosecond laser can be applied to fabricate micro-gratings on the surface of the specimen without a mask, which provides an alternative way of grating fabrication, and its corresponding research is of significant importance. Recently, nanosecond laser was reported to fabricate micro-scale periodic structures. For example, Gedvilas et al. investigated the formation of regular gratings of ripples using a nanosecond laser [26]. Luo et al. fabricated multimode and single-mode Fiber Bragg Gratings (FBGs) using a 355 nm laser [27]. Xia et al. etched subwavelength periodic ripples on the surface of silica films induced by nanosecond laser pulses [28]. Kishimoto et al. proposed to fabricate micro model grid for various Moiré methods by femtosecond laser exposure. The parallel lines and cross grid were fabricated directly on the surface of polished steel specimens, and the spacing between neighboring lines of the model grid is 10 or 20 μm [29]. When gratings are fabricated on specimens using a nanosecond laser for mechanical testing, processing parameters on different materials are not the same, which is a significant shortcoming for material testing. 

In the present study, fabrication of micro-scale parallel lines and cross grid are implemented using a nanosecond laser. Various materials are tested for parameter studying. When a thin layer of Al film is first deposited on the specimen, the fabricated gratings are of high quality and the processing parameters can be optimized for different material tests. The fabrication of parallel and cross gratings is demonstrated with a tensile specimen and a V-notched specimen, and the strain fields are calculated by the GPA method. 

## 2. Methodology

### 2.1. Nanosecond Laser Processing and Gratings

**Figure 1:**
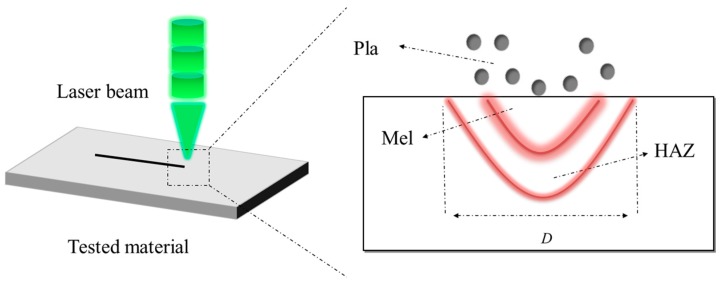
Schematic diagram of nanosecond laser processing.

Pulsed laser interaction is a material forming or modification process. As shown in Figure 1, when a material is irradiated by a high-energy laser beam, the temperature of the spotted surface increases sharply due to the absorption of the laser energy. The material begins to melt when the temperature of the spotted area reaches its melting point. If the temperature continues rising and reaches the latent heat of material ablation with further absorption of laser energy, the material will begin to evaporate. Generally, the energy of a nanosecond laser beam has a Gaussian distribution. There are three regions when the high-energy laser irradiates to the material (Figure 1): (1) a plasma region (Pla) with high temperature and density formed on the surface of the material, (2) a melting region (Mel) in the center of the spotted material, and (3) a heat affected zone (HAZ) under the melting region [30,31].

The damage threshold of a material irradiated by a laser beam is one of its intrinsic properties. When a single-pulse laser irradiates on a material, the damage of the material is mainly associated with the energy of the laser. The relationship between the laser intensity (*I*_0_) on the surface of the specimen and its single-pulse energy *E* is [32],

(1) $$I_{0}\;=\;\frac{2E}{\pi \omega_{0}^{2}}$$

where *ω*_0_ is the waist radius of the laser beam (μm). And the etching diameter can be determined as,

(2) $$D^{2}\;=\;2\omega_{0}^{2}ln\left(\frac{I_{0}}{I_{th}}\right)$$

where *D* indicates the etching diameter and *I_th_* is the threshold of energy fluence (J/cm^2^). Substituting Equation (1) into Equation (2) gives,

(3) $$D^{2}\;=\;2\omega_{0}^{2}lnE+2\omega_{0}^{2}ln\left(\frac{2}{\pi \omega_{0}^{2}I_{th}}\right)$$

Ablation takes place in areas where the laser fluence exceeds the ablation threshold. The width of line area is approximated to the ablated line width when a single laser beam is used. When parallel gratings are fabricated using a nanosecond laser, the frequency *f* and pitch *p* of the gratings can be expressed as,

(4) $$f\;=\;\frac{1}{p}$$

(5) p = D+d

where *d* is the distance between two adjacent lines and *D* represents the line width. Theoretically, the grating frequency is higher when *D* and *d* are smaller.

### 2.2. GPA

The displacement and strain can be obtained by the measurement of the grating alterations. A grating image of gray-function can be described as a Fourier series,

(6) $$I\left(r\right)\;=\;\underset{f}{\sum }A_{f}e^{2i\pi f\cdot r}$$

where *I*(*r*) is the intensity in the image at position *r*, and *A_f_* represents the Fourier component. To describe the variations of contrast and fringe position in the image, the Fourier component *A_f_* is related to the position *r*,

(7) $$I\left(r\right)\;=\;\underset{f}{\sum }A_{f}\left(r\right)e^{2i\pi f\cdot r}$$

The local Fourier components are obtained by filtering from the Fourier spectrum, and *I_f_* is therefore given by,

(8) $$I_{f}\left(r\right)\;=\;A_{f}\left(r\right)e^{2\pi if\cdot r}$$

Assuming that there is a displacement *u*(*r*),

(9) r→r−u(r)

then Equation (6) becomes,

(10) $$I_{f}^{'}\left(r\right)\;=\;A_{f}\left(r\right)e^{2\pi if\cdot \left\{r-u\left(r\right)\right\}}$$

The phase of these local Fourier components or the geometric phase *P_f_*(*r*) is directly related to the component of the displacement field *u*(*r*) in the direction of the reciprocal lattice vector *f*,

(11) $$P_{f}\left(r\right)\;=\;-2\pi fu\left(r\right)$$

The solution of the displacement is,

(12) $$u\left(r\right)\;=\;-\frac{1}{2\pi }\left[p_{f1}\left(r\right)\frac{1}{f_{1}}+p_{f2}\left(r\right)\frac{1}{f_{2}}\right]$$

Therefore, the displacements can be derived as,

(13) $$\left(\begin{array}{c} u_{x}\\ u_{y} \end{array}\right)\;=\;-\frac{1}{2\pi }{\left(\begin{array}{cc} f_{1x} & f_{1y}\\ f_{2x} & f_{2y} \end{array}\right)}^{-1}\left(\begin{array}{c} P_{f_{1}}\\ P_{f_{2}} \end{array}\right)$$

where the subscripts *x* and *y* represent the *x* and *y* directions respectively. Plane strain can be written as,

(14) $$\varepsilon_{xx}\;=\;\frac{\partial u_{x}}{\partial x},\;\varepsilon_{yy}\;=\;\frac{\partial u_{y}}{\partial y},\;\varepsilon_{xy}\;=\;\frac{1}{2}\left(\frac{\partial u_{x}}{\partial y}+\frac{\partial u_{y}}{\partial x}\right)$$

## 3. Experimental Setup and Tested Samples

**Figure 2:**
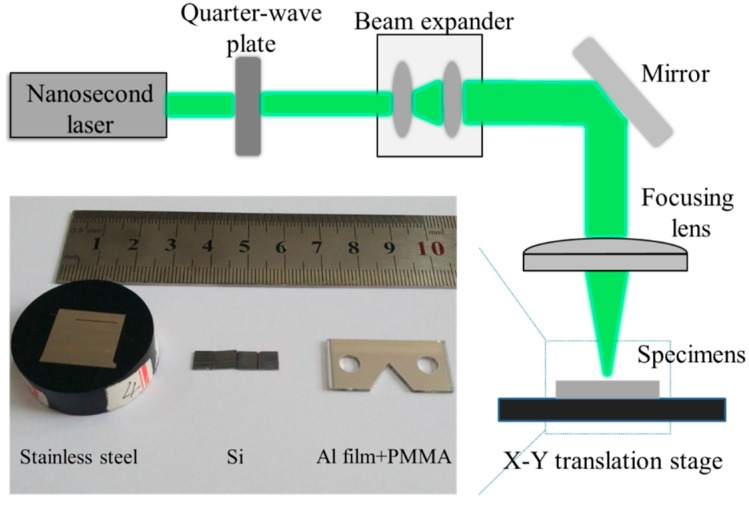
Schematic diagram of a nanosecond laser processing system and the image of specimens.

A schematic diagram of a nanosecond laser processing system is shown in Figure 2. The system includes an Nd: YVO_4_ laser (Continuum, PPII8000, Felles Photonic, Tianjin, China), a quarter-wave plate, a beam expander, a focusing lens, and an *X*-*Y* translation stage. The nanosecond laser has a wavelength of 532 nm, pulse width of 500 ns, pulse frequency of 1 kHz, and maximum pulse energy of 1 J. During the experiment, the laser beam first passes through a quarter-wave plate to enable circular polarization and then is expanded by a beam expander before it is reflected by a mirror. Subsequently, the laser beam is guided by a focusing lens to reach the test specimen. The laser frequency and scanning speed are constant, being 1 kHz and 10 mm/s, respectively.

The test samples include Si, quartz glass, stainless steel, and Al-film coated specimens with the purpose of parameter studying. The stainless steel specimen was ground and polished carefully before the nanosecond laser processing. Al film was deposited by current heating and its thickness is about 2 μm. Some of the tested specimens are shown in Figure 2. In order to obtain etching parameters of the target material when a pulse laser beam irradiates on the specimen, lines were etched by the nanosecond laser system with different pulse energy. Micro-structural morphology and widths of the etched areas were observed and measured by an optical microscopy and a scanning electron microscope (SEM, Hitachi, Tokyo, Japan). Suitable parameters were chosen to fabricate micro-scale gratings.

**Figure 3:**
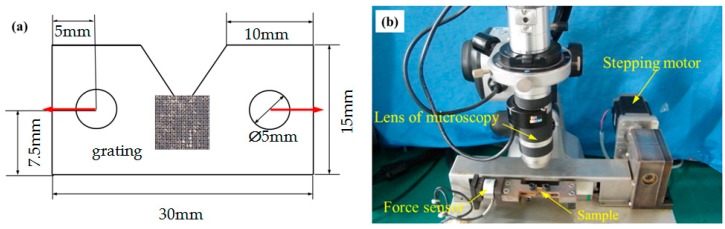
(**a**) Schematic of the polymethyl methacrylate (PMMA) specimen and (**b**) the experimental setup including an optical microscope and a tensile loading stage.

For applications, a tensile test was conducted on a rubber specimen with a dimension of 100 mm × 20 mm × 5 mm (thickness of 5 mm). An aluminum film was firstly deposited on the specimen surface, followed by parallel gratings fabricated by the nanosecond laser. The micro-scale gratings at the center of the specimen was traced and captured under different loads. Second, a micro-scale cross grating was fabricated to an Al film–coated polymethyl methacrylate (PMMA) specimen with a V-shaped notch. The dimensions of the specimen are shown in Figure 3a. The thickness of the specimen is 1 mm. Tensile tests were also performed, and the micro-scale gratings at the crack tip were traced and captured for the strain calculation. The field of view can be fixed by a small tensile loading stage during the tests with bidirectional loads at the same speeds, as shown in Figure 3b. The displacement can be controlled automatically with a stepping motor. The resolutions for the force and the displacement were 1 N and 6 μm, respectively. 

## 4. Experimental Results and Discussion

### 4.1. Parameter Studying of Single-Line Etching

**Figure 4:**
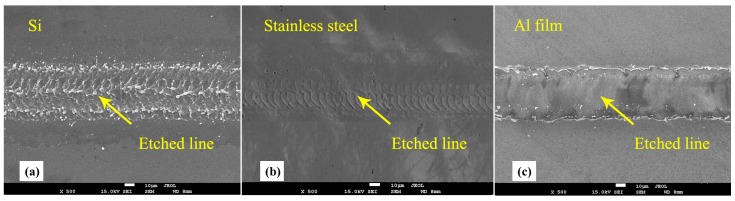
Scanning electron microscopy (SEM) images of single line etched by nanosecond laser: (**a**) Si, (**b**) stainless steel and (**c**) Al film on quartz glass. The etching energy is 38.6 μJ, 20.0 μJ, and 31.5 μJ.

For the parameter study of single-line etching by the nanosecond laser, different materials were used, and the pulse energy of the laser was changed. Figure 4 shows SEM images of single line etched by nanosecond laser. It can be seen that the morphology of laser-processed area is changed. Figure 4a clearly shows evaporation of material along the line when the laser beam is irradiated. Figure 4b shows the spots of laser pulse along the scanning line. Figure 4c indicates that the Al film has been etched, and the etched line on the Al film is clear and continuous. The results demonstrate the feasibility of line fabrication using a nanosecond laser on different materials.

**Figure 5:**
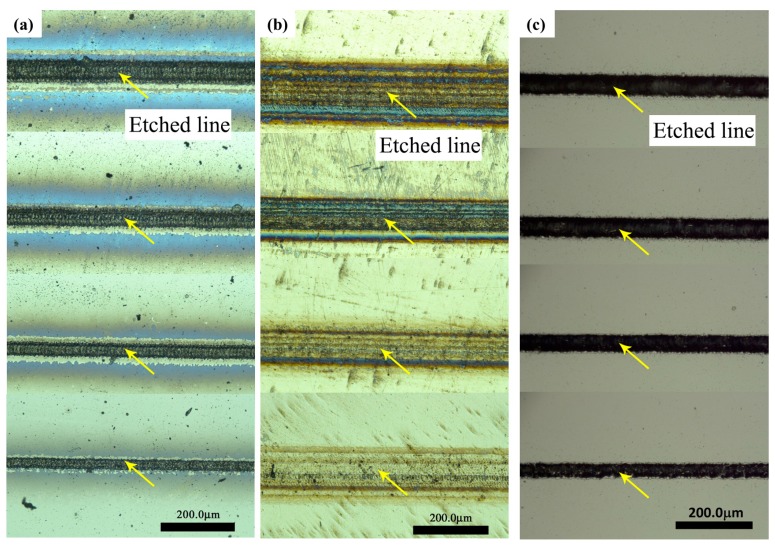
Optical images of straight lines etched by nanosecond laser: (**a**) Si, (**b**) stainless steel, and (**c**) Al film. The etching energy is about 80 μJ, 51 μJ, 39 μJ, and 17 μJ from top to bottom. The arrows indicate the etched lines.

Figure 5a–c shows etched lines on the specimens of Si, stainless steel, and Al film, respectively. The etching energy were not the same; the values were about 80, 51, 39, and 17 μJ from top to bottom. The optical images apparently show the straight lines etched by the nanosecond laser, and the material on each straight line has been ablated. In addition, the line width and the HAZ decrease with the decreasing pulse energy. The line width of Si reaches about 30 μm when the pulse energy is about 17 μJ. It can also be seen that the surface of the Al film is cleaner and smoother than the polished surface. Furthermore, the HAZ of each line is very small, thus the definition and quality of each line are better.

**Figure 6:**
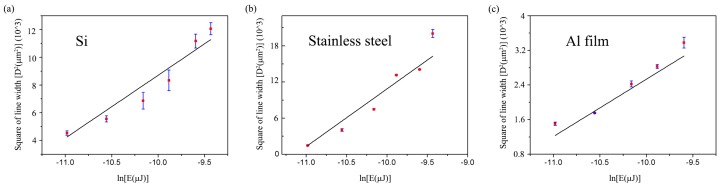
Linear fitting of measured data between the square of line width and logarithm of single pulse energy: (**a**) Si, (**b**) stainless steel, and (**c**) Al film.

According to Equation (3), the relations between the square of line width and the logarithm of single pulse energy are shown in Figure 6. The measured data is linearly fitted. The slope of each line is the value of the waist radius ($2\omega_{0}^{2}$). The waist radius of Al film is 25.7 μm, which is much smaller than that of Si and stainless steel. As a result, the line width of Al film is less than that of Si and stainless steel, as indicated in Figure 5. The threshold of single-pulse energy (*E_th_*) can be obtained when *D* = 0 based on Equation (3). Thus, the threshold of energy density (*I_th_*) can be calculated according to Equation (1). The calculated results of Si, stainless steel, and Al film are listed in Table 1.

Experiments on different materials etching with a nanosecond laser demonstrate that the etching parameters of lines are different. For example, materials with high melting point, like ceramic and quartz glass, require large pulse energy to etch lines. At the same time, the waist radii of Si and stainless steel are relatively large, resulting a relatively large width of etched line, so they are not suitable for the fabrication of micro-scale gratings. Furthermore, the etched line usually has many discontinuities, thus line quality is not high. Roughness and marks on the surface of polished specimens will lower the quality of the etched lines even with fine grinding and careful polishing. Therefore, film deposition on a specimen is recommended before the laser processing. There are two benefits: first, processing parameters of line etching are the same with different materials, which is convenient for operation; second, line quality is good with high contrast, small HAZ, and small roughness.

**Figure 7:**
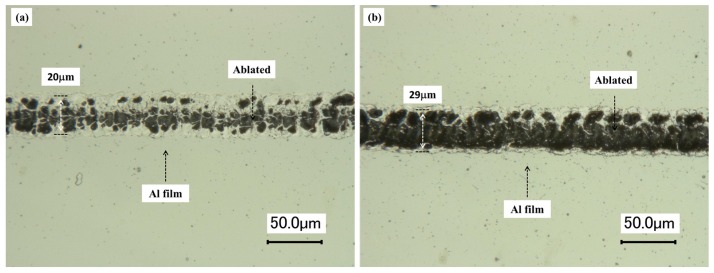
Optical images of straight line etched by nanosecond laser: (**a**) etching energy is 6.8 μJ, and (**b**) etching energy is 9.8 μJ.

**Figure 8:**
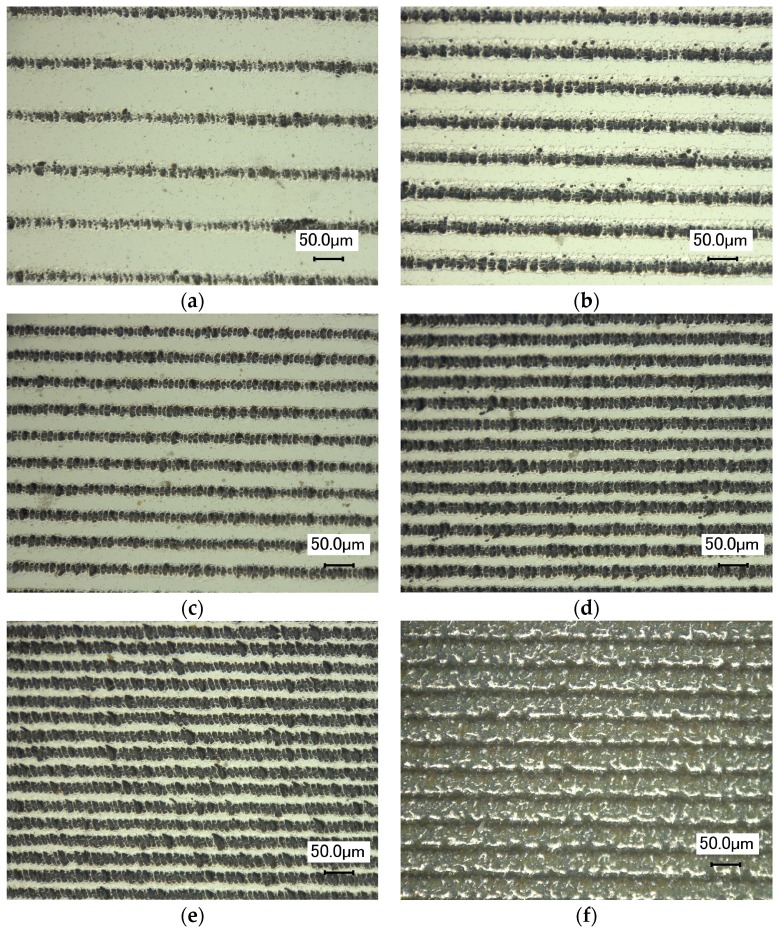
Optical images of gratings fabricated on Al film: (**a**) *d* = 50 μm, (**b**) *d* = 38.2 μm, (**c**) *d* = 28.1 μm, (**d**) *d* = 13.9 μm, (**e**) *d* = 10 μm, and (**f**) *d* = 0 μm. The frequency of gratings is (**a**) 10 lines/mm, (**b**) 15 lines/mm, (**c**) 20 lines/mm, (**d**) 25 lines/mm, (**e**) 30 lines/mm, and (**f**) 35 lines/mm. The energy of laser pulse is 9.8 μJ.

It should be noted that the quality of the etched line is not high when the threshold pulse energy is used, as shown in Figure 7a. The energy of laser pulse is 6.8 μJ, which is the threshold pulse energy of Al film. There remains a trail of Al marks along the line of laser ablation and obviously the ablation process is not stable. As a result, the boundary and contrast of the etched line is not clear. As shown in Figure 8b, the energy of laser pulse is 9.8 μJ, which is slightly higher than the threshold pulse energy of Al film. The width of the etched line is 29 μm, which is larger than that in Figure 8a. However, Al film has been ablated almost completely along the laser path and the etched line is clear, continuous, and of high contrast. Though higher energy of laser pulse can be used for line etching, the line width is larger, which is not beneficial for subsequent fabrication of high frequency gratings. It should be pointed out that 9.8 μJ is the optimal energy of laser pulse (i.e., the optimum fluence is 9.5 J/mm^2^ with the waist radius of the laser beam 25.7 μm). Therefore, the above optimum parameters are recommended for line etching of 2 μm thickness Al film. The line width is 29 μm. 

### 4.2. Gratings

Parallel gratings were fabricated using the above mentioned optimal parameters for single line etching. Figure 8 shows the SEM images of gratings fabricated on Al film with varying *d* from 50 μm to 0 μm. The frequency of Figure 8a–f is from 10 lines/mm to 35 lines/mm. The lines were fabricated by scanning from left to right and from top to bottom. Figure 8a shows parallel grating structures when *d* = 50 μm. The width of ablated line is larger than that of the Al-film strip. As can be seen, the boundary of each line is clear and the contrast between the ablated area and Al film is obvious. Figure 8b–f shows parallel grating structures when d decreases. There are more ablated lines in the same field of view with decreasing width of the Al-film strip. Figure 8f shows the Al film in the field of view is entirely ablated when *d* = 0, and the ablated lines are connected, which is difficult to be distinguished.

**Figure 9:**
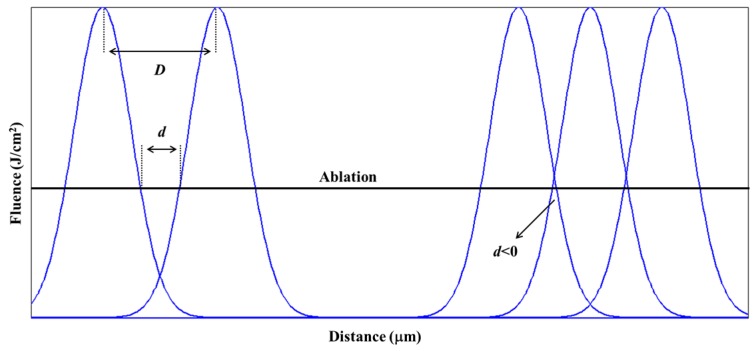
Schematic diagram of etching with multi laser beams with respect to distance.

The above experimental phenomenon can be explained as shown in Figure 9. Assuming that the horizontal dash line is the threshold fluence for Al film ablation, the peak fluence of the laser beam is higher than the ablation threshold of Al film, and thus the center area is ablated. The oblique line area of laser pulse is equal to the ablated line width *D*, which is constant when the energy of laser pulse is fixed (9.8 μJ). However, the outer edges are subject to lower irradiation intensity, and thus, a strip of Al film remains. Apparently, the subsequent laser pulse has no influence on the former ablated line when *d* > 0. As indicated in Equations (4) and (5), the frequency of gratings is higher when the distance between two adjacent laser pulses is smaller. The increasing frequency of gratings can be realized by decreasing *d*, as shown in Figure 8a–e. However, the distance between two adjacent laser pulses cannot be negative. The ablated areas are overlapped as shown in the right-three laser pulses in Figure 9. Although the grating frequency is high, the etched lines are not continuous, and the quality of the grating is poor since the subsequent laser pulse has a severe influence on the former ablation area. Therefore, gratings of parallel lines fabricated by nanosecond laser will affect each other if the distance between adjacent lines is too small. The highest frequency of parallel gratings is about 30 lines/mm with line width of 29 μm and the distance between two adjacent laser pulses of 10 μm. 

## 5. Applications

### 5.1. Deformation Measurement during a Tensile Test

**Figure 10:**
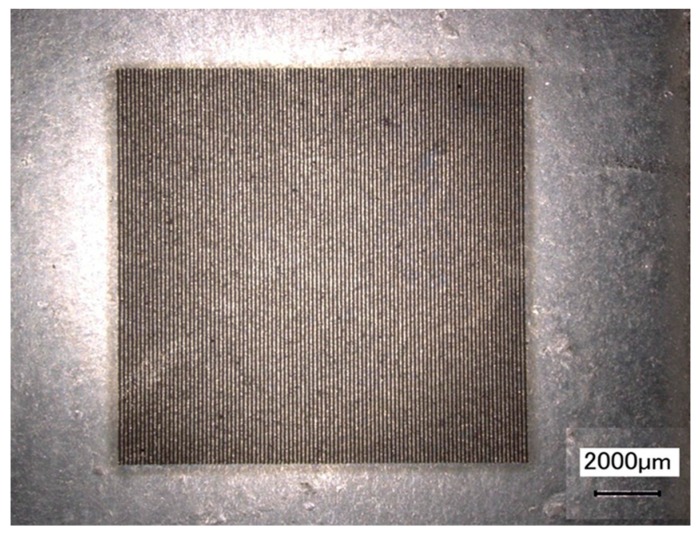
Parallel gratings fabricated on a rubber tensile specimen before load.

**Figure 11:**
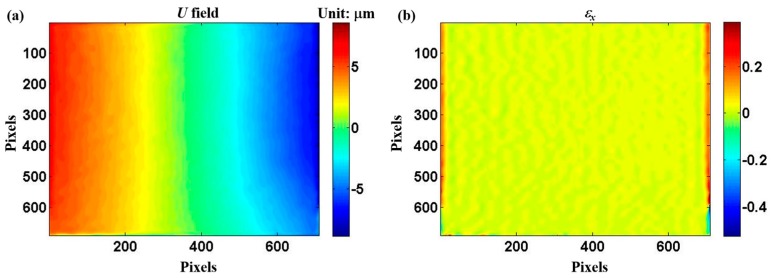
Calculated displacement and strain fields in horizontal direction: (**a**) *U* and (**b**) *ε_x_*. One pixel represents 0.55 μm. The tensile load is under 52.9 N.

As a verification study, a rubber tensile specimen was coated with a thin layer of Al film, followed by the fabrication of micro-scale gratings on the film. The thickness of the Al film was about 2 μm, which had little influence on the mechanical properties of the substrate. The grating frequency was 10 lines/mm. Figure 10 shows the images of parallel gratings before load. The images of parallel gratings after load are not shown since they are almost the same. The calculated displacement and strain fields under 52.9 N load are shown in Figure 11. It can be seen that the displacement on the vertical center line is almost zero; the left and right parts are symmetric on the vertical center line. The calculated strain field has an even distribution and the average value of the strain is about 0.01. 

**Figure 12:**
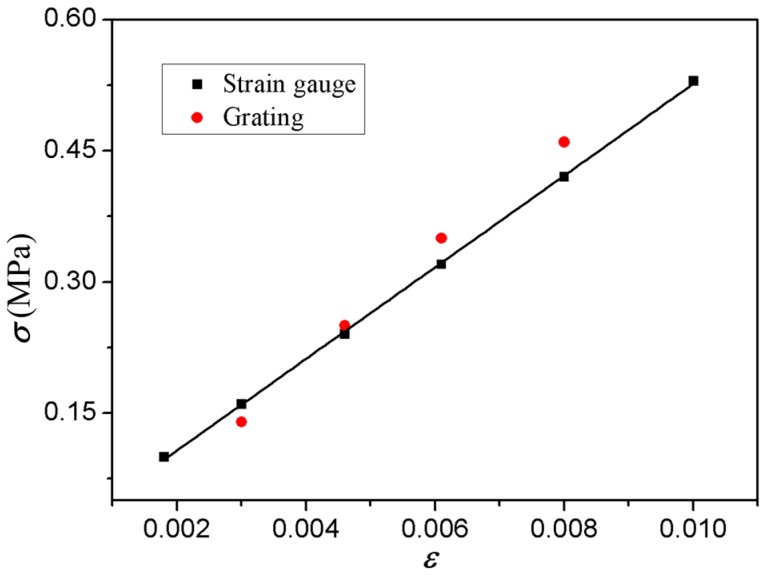
Stress-strain relation measured by strain gauge and gratings.

Strain gauges were used to measure the strains under different loads simultaneously, and the measured data and calculated strains using GPA were plotted, as shown in Figure 12. The stress-strain data measured by GPA agrees well with that by strain gauges, which verifies the applicability of the proposed fabrication method for the micro-scale gratings in GPA measurements.

### 5.2. Deformation Measurement of the Crack Tip

**Figure 13:**
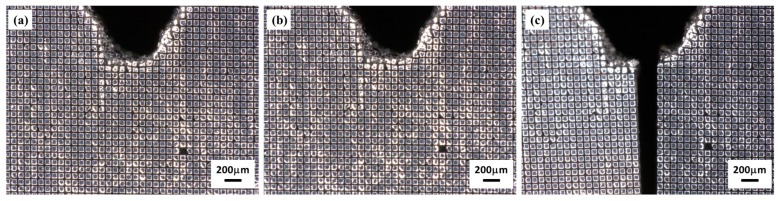
Gratings on the PMMA specimen with a V-shape notch: (**a**) before load, (**b**) under 49.0 N load, and (**c**) under load 73.5 N.

**Figure 14:**
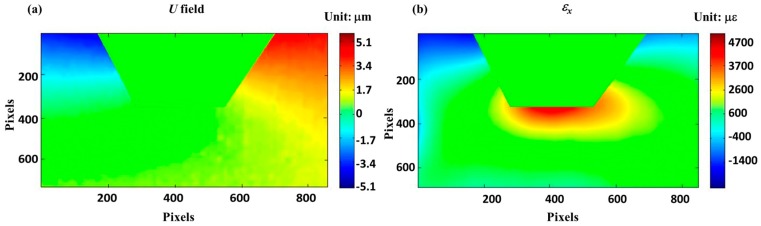
Calculated displacement and strain fields in horizontal direction under load 49.0 N: (**a**) *U* and (**b**) *ε_x_*. One pixel represents 0.55 μm.

A PMMA specimen with a V-shape notch was also deposited with a thin layer of Al film and followed by fabrication of cross gratings on the film. The thickness of Al film was about 2 μm. The frequency of cross gratings was 10 lines/mm and the area was about 5 mm × 5 mm. The specimen was put on the tensile loading stage, and the cross gratings at the crack tip were captured under progressive loads. Figure 13 shows the images of gratings near the V-shape notch before load, under 49.0 N load, and after crack propagation. It can be seen that the cross gratings can be fabricated at the boundary of the notch and the gratings are of high quality. In addition, the adhesion between the Al film and the tested sample is good enough to ensure that the pattern sticks well to the sample, even when the specimen begins to crack at the notch tip. The calculated displacement and strain fields at the crack tip were successfully obtained at a tensile load of 49.0 N, as shown in Figure 14. The displacement and strain fields are symmetric perpendicular to the loading direction, and the tensile strain is concentrated at the crack tip, which agrees well with the theory of fracture mechanics [33].

## 6. Conclusions

In this paper, the fabrication and optimization of micro-scale gratings formed by nanosecond laser etching are investigated. The mechanism of nanosecond laser processing and the geometric phase analysis are theoretically analyzed. The factors influencing the fabrication process including laser energy, laser fluence, and ablation threshold of material are experimentally studied by considering the ablated line width. Different materials are tested for the purpose of parameter study, and Al film deposition on specimen before laser processing is proposed for the fabrication of high-quality gratings. In addition, the micro-scale gratings are fabricated on specimens and experimentally verified by performing tensile tests. Some important conclusions can be summarized as follows:

(1) Al film deposition on specimen before laser processing is proposed for fabrication of micro-scale gratings with three main benefits. First, easy operation with the same processing parameters and without a mask. Second, wide applicability to different materials, such as Si, metal, ceramic, composite, etc. Third, high-quality of gratings with high contrast, small HAZ and small roughness.
(2) The energy of laser pulse is optimized for clear line etching on the Al film. The optimal energy of laser pulse is 9.8 μJ, and the optimum fluence is 9.5 J/mm^2^ with the waist radius of the laser beam 25.7 μm. Parallel gratings are fabricated. The results indicate that gratings of parallel lines fabricated by nanosecond laser will affect each other if the distance between adjacent lines is too small. The highest frequency of parallel gratings is about 30 lines/mm with line width of 29 μm, and the distance between two adjacent laser pulses being of 10 μm.
(3) The verification tests prove that the applicability of the proposed fabrication method for the micro-scale gratings in GPA measurements. Moreover, the micro-scale gratings can be fabricated on areas of interest, such as the crack tip, for deformation measurements. The adhesion between the Al film and the tested sample is good enough to ensure that the pattern sticks well to the sample.
(4) The proposed fabrication method of gratings suffers from a few defects. For instance, the minimum line width of gratings is about 10 μm due to the spatial resolution of the nanosecond laser, and thus the frequency of fabricated gratings is relatively low, typically 10–30 lines/mm. If a femtosecond laser is used, higher frequency of gratings can be fabricated since it has higher spatial resolution and pulse energy.

## Figures and Tables

**Table 1:** Calculated *ω*_0_, *E_th_*, and *I_th_* of Si, stainless steel, and Al film.

Specimens	*Ω*_0_ (μm)	*E_th_* (μJ)	*I_th_* (J/mm^2^)
Si	52.0	8.71	2.05
Stainless Steel	75.6	17.0	1.89
Al Film	25.7	6.8	6.56
